# Ribosomal S6 Kinase 2 (RSK2) Maintains Genomic Stability by Activating the Atm/p53-Dependent DNA Damage Pathway

**DOI:** 10.1371/journal.pone.0074334

**Published:** 2013-09-23

**Authors:** Han Chi Lim, Li Xie, Wei Zhang, Rong Li, Zhong-Can Chen, Guang-Zhi Wu, Shu-Sen Cui, Eng King Tan, Li Zeng

**Affiliations:** 1 Neural Stem Cell Research Lab, Research Department, National Neuroscience Institute, Singapore, Singapore; 2 Experimental Therapeutics Centre, c/o Biomedical Sciences Institutes (BMSI), A*STAR, Singapore, Singapore; 3 Department of Pharmacology, Yong Loo Lin School of Medicine, National University of Singapore, Singapore, Singapore; 4 Department of Hand-surgery, China-Japan Union Hospital, Jilin University, Changchun City, Jinlin Province, People's Republic of China; 5 Research Department, National Neuroscience Institute, Singapore, Singapore; 6 Neurology Department, National Neuroscience Institute, Singapore, Singapore; 7 Neuroscience & Behavioral Disorders program, DUKE-NUS Graduate Medical School, Singapore, Singapore; National University of Singapore, Singapore

## Abstract

Ribosomal S6 Kinase 2 (RSK2) is a member of the p90^RSK^ family of serine/threonine kinases, which are widely expressed and respond to many growth factors, peptide hormones, and neurotransmitters. Loss-of function mutations in the *RPS6KA3* gene, which encodes the RSK2 protein, have been implicated in Coffin-Lowry Syndrome (CLS), an X-linked mental retardation disorder associated with cognitive deficits and behavioral impairments. However, the cellular and molecular mechanisms underlying this neurological disorder are not known. Recent evidence suggests that defective DNA damage signaling might be associated with neurological disorders, but the role of RSK2 in the DNA damage pathway remains to be elucidated. Here, we show that Adriamycin-induced DNA damage leads to the phosphorylation of RSK2 at Ser227 and Thr577 in the chromatin fraction, promotes RSK2 nuclear translocation, and enhances RSK2 and Atm interactions in the nuclear fraction. Furthermore, using RSK2 knockout mouse fibroblasts and RSK2-deficient cells from CLS patients, we demonstrate that ablation of RSK2 impairs the phosphorylation of Atm at Ser1981 and the phosphorylation of p53 at Ser18 (mouse) or Ser15 (human) in response to genotoxic stress. We also show that RSK2 affects p53-mediated downstream cellular events in response to DNA damage, that RSK2 knockout relieves cell cycle arrest at the G2/M phase, and that an increased number of γH2AX foci, which are associated with defects in DNA repair, are present in RSK2-deficient cells. Taken together, our findings demonstrated that RSK2 plays an important role in the DNA damage pathway that maintains genomic stability by mediating cell cycle progression and DNA repair.

## Introduction

Coffin-Lowry syndrome (CLS) is an X-linked mental retardation disorder caused by mutations in the *Rps6ka3* gene, which encodes ribosomal S6 kinase (RSK) 2 [1]. This syndrome is characterized by psychomotor, growth, and cognitive retardation, as well as facial, hand, and skeletal anomalies [2].

CLS patients have markedly reduced cerebellar and hippocampal volumes compared to healthy controls [3]. RSK2 plays a key role in this neurological disorder. In the adult mouse brain, RSK2 is highly expressed in regions with high synaptic activity, including the cerebellar Purkinje cells and the pyramidal cells of the CA3 hippocampal region [4]. Studies have shown that the functional impairment of neurotransmission and plasticity due to AMPAR dysfunction may contribute to the cognitive deficit observed in RSK2 knockout (KO) mice [5]. In addition, loss of RSK2 function decreases neurogenesis during cerebral cortex development [6]. These data suggest that RSK2 plays an important role in learning and memory in both humans and mice and that RSK2 deficiency might lead to cognitive and behavioral dysfunction.

Several lines of evidence have linked DNA damage and repair systems to neurological disorders. DNA damage can be caused by exogenous or endogenous factors, such as ionizing radiation (IR), chemotherapeutic drugs, and stalled replication forks [7]. Upon exposure to DNA-damage reagents, mammalian cells trigger a sequence of multi-component biochemical reactions to maintain genome integrity. At the core of the signaling network are PI3 kinase-like kinases (PIKKs), including Atm, Atr and DNA-PKcs [8]. Atm and Atr are recruited to nuclear foci by the MRN (Mre11-Rad50-NBS) complex [9], where they phosphorylate proteins such as p53, Chk1, Chk2, and H2AX to activate cell cycle checkpoints and/or induce apoptosis [10].

Patients with Ataxia Telangiectasia (A-T) and Seckel Syndrome-1 (SCKL1) exhibit severe cerebellar degeneration, microcephaly and mental retardation, which result from deficiencies in Atm and Atr, respectively [11]–[12]. Furthermore, growing evidence links DNA damage to cognitive impairment in experimental animals and patients receiving genotoxic chemotherapeutic drugs [13]–[14]. For instance, data from a longitudinal study of breast cancer patients who were evaluated using structural and functional Magnetic Resonance Imaging (MRI) before treatment and 1 and 12 months after treatment suggest a pattern of reduced activation in frontal areas during a working memory task [15]. Recently, RSK2 was reported to directly phosphorylate histone H2AX. The incorporation of phosphorylated H2AX in chromatin is an indicator of DNA damage, suggests a possible role for RSK2 in maintaining chromatin stability [16]. In addition, RSK2 activates p53 *in vitro* and *in vivo*, and co-localizes with p53 in the nucleus [17]. However, the primary function of RSK2 in the DNA damage pathway remains unclear.

While investigating the role of RSK2 in the DNA damage response and DNA repair, we observed the phosphorylation of RSK2 at Ser227 and Thr577 in response to Adriamycin-induced DNA damage. We demonstrated that RSK2 activates the Atm/p53-dependent DNA damage pathway and that RSK2 also interacted and co-localized with Atm in the nucleus upon DNA damage. RSK2 participated in the DNA damage response by affecting p53-controlled cell cycle progression and DNA repair mechanisms. The absence of RSK2 relieved cell cycle arrest at the G2/M phase, increasing the number of γH2AX foci (which are associated with DNA repair) in both RSK2 knockout (KO) mouse embryonic fibroblasts (MEFs) and CLS patient fibroblast cells. Hence, our study has uncovered a novel role for RSK2 in the DNA damage pathway and provided a link between CLS, a neurological disorder caused by mutated RSK2, and defects in the DNA damage response.

## Results

### Genotoxic stress results in RSK2 phosphorylation at Ser227 and Thr577

**Figure 1:**
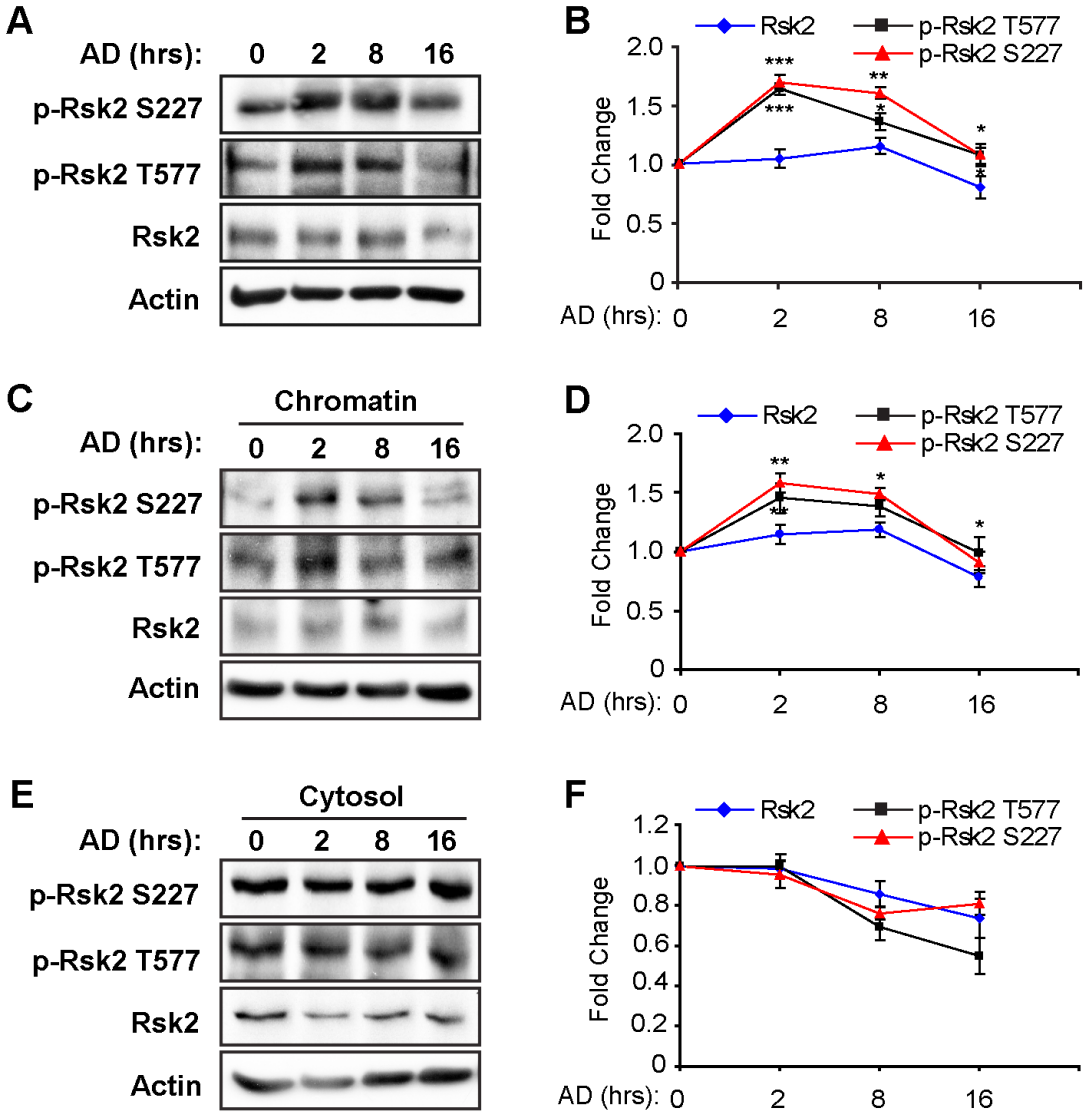
Genotoxic stress results in RSK2 phosphorylation at Ser227 and Thr577. A. MEFs were treated with 0.5 µM Adriamycin (AD) for specific periods of time to induce DNA lesions. Protein levels of total RSK2 and RSK2 phosphorylated at Ser227 and Thr577 were analyzed by western blot. B. Quantitation of RSK2 phosphorylation at Ser227 and Thr577. The value of phospho-RSK2 at time zero in the absence of AD was set at 1.0. Increased levels of phospho-RSK2 were observed 2 hr after exposure of the MEFs to AD. C. RSK2 is associated with chromatin and activated by a DNA damaging reagent. MEFs were treated with 0.5 µM AD for various time points, followed by fractionation into the cytosolic and chromatin compartments. Western blot was performed to analyze the protein levels of total RSK2 and phospho-RSK2. D. Quantitation of RSK2 phosphorylation at Ser227 and Thr577 in the chromatin fraction. The value of phospho-RSK2 at time zero in the absence of AD was set at 1.0. An increased level of phosphorylated RSK2 was observed in the chromatin fraction, indicating an increased association between activated RSK2 and chromatin under genotoxic stress, whereas the level of cytosolic phospho-RSK2 remained the same as shown in E and F. For each experiment, at least three replicates were performed, and similar results were obtained. Representative results from one experiment are shown. All data are shown as the mean plus or minus the standard deviation of the mean (mean ± SD). A significant difference was defined as *P<0.05, **P<0.01, and ***P<0.001 compared to the control.

To maintain genomic integrity, DNA damage in mammalian cells rapidly activates a number of specific signaling pathways that initiate cell cycle arrest and subsequent DNA repair. To examine the potential role of RSK2 in the DNA damage response, we treated low-passage, non-immortalized MEFs with DNA-damaging agents for various lengths of time, and then, we detected the phosphorylation of RSK2 in response to the DNA damage. DNA damage was initially induced by treatment with Adriamycin (AD), a chemotherapeutic agent used to treat a wide variety of cancers. AD is a topoisomerase II inhibitor that has been shown to induce DSBs and SSBs by activating both Atm and Atr [18]. We found that after 2 hr of AD treatment, RSK2 was phosphorylated at Ser227 (∼1.6-fold) in the N-terminal kinase domain (NTKD) and Thr577 (∼1.6-fold) in the C-terminal kinase domain (CTKD). RSK2 remained phosphorylated for up to 16 hr after AD treatment (Fig. 1A and B). The levels of RSK2 expression remained constant during the AD treatment.

Several DNA damage response and DNA repair proteins, such as DNA-PK, Rad51, and HMGB1, associate with chromatin in response to DNA damage [19]–[20]. To test whether RSK2 associates with chromatin after DNA damage, we treated MEFs with 0.5 µM of AD and then isolated the chromatin-associated proteins [21]. We found an increased amount of RSK2 phosphorylated at Ser227 and Thr577 in the chromatin fraction after treating with AD for 2 hr (Fig. 1C and D), but this effect was not observed in the cytosolic fraction (Fig. 1E and F). This indicates that activated RSK2 associates with chromatin in response to DNA damage.

### RSK2 deficiency impairs the Atm/p53-dependent DNA damage pathway

**Figure 2:**
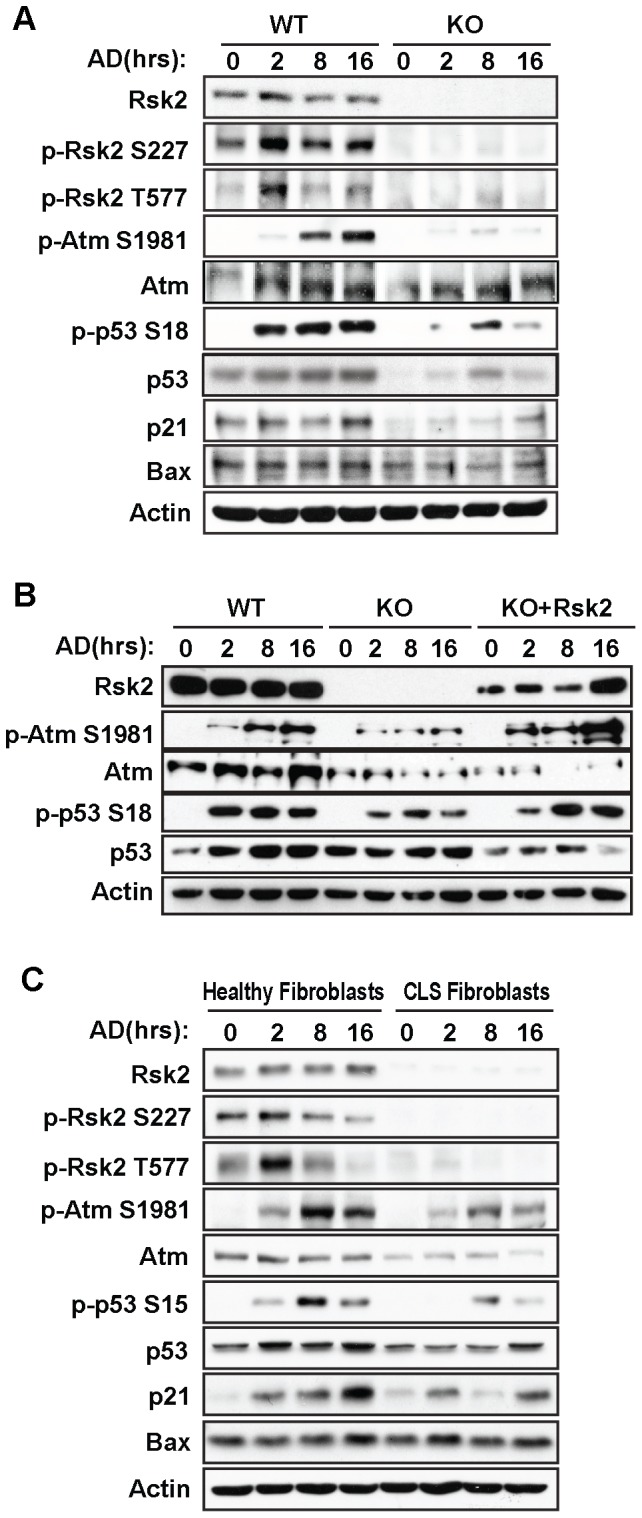
RSK2 activates the Atm/p53-dependent DNA damage pathway under genotoxic stress. A. RSK2 activates the Atm/p53-dependent DNA damage pathway under genotoxic stress in MEFs. RSK2 WT and KO MEFs were isolated from E14.5 mouse embryos. Cells were treated with 0.5 µM AD for 2 hr, 8 hr, or 16 hr. The cell lysates were then analyzed by western blot against Atm, phospho-Atm(Ser1981), and DNA damage response target genes downstream of p53, including phospho-p53(Ser18), Bax and p21. The RSK2 KO MEFs shows very little activation signal due to the RSK2 deficiency, and this leads to decreased phosphorylation of Atm at Ser1981, decreased p53 protein stability and transactivation, and decreased levels of p21 expression. Total RSK2 and β-actin were used as internal controls to confirm the RSK2 knockout and equal protein loading. B. Overexpression of RSK2 in the RSK2 KO MEFs rescued Atm and p53 activation. RSK2 WT and KO MEFs were isolated from E14.5 mouse embryos. RSK2 was overexpressed in RSK2 KO MEFs, and a control plasmid was overexpressed in the WT and KO RSK2 cells. Cells were treated with 0.5 µM AD for 2 hr, 8 hr or 16 hr. The cell lysates were then analyzed by western blot for the expression of Atm, phospho-Atm(Ser1981), p53 downstream targets, and phospho-p53 (Ser18). C. RSK2 activates the Atm/p53-dependent DNA damage pathway under genotoxic stress in human fibroblasts. Healthy fibroblasts (GM09621) and RSK2-deficient fibroblasts from CLS patients (GM03321) were cultured and exposed to 0.5 µM AD for various times. Western blot analysis was conducted using phospho-specific antibodies for Atm and p53. Total RSK2 and β-actin were used as internal controls to confirm RSK2 knockout and equal protein loading.

Our data show that RSK2 is phosphorylated at Ser227 and Thr557 in response to genotoxic stress, which is known to cause DSBs and SSBs. To determine the role of RSK2 in the DNA damage response, we first examined whether the absence of functional RSK2 would affect the activation of the Atm/p53-dependent DNA damage pathway, which is known to trigger the DNA damage signaling network [10]. We cultured RSK2 wild type (WT) and knockout (KO) MEFs and treated the cells with 0.5 µM AD for 2 hr, 8 hr, or 16 hr. We found that AD-induced DNA damage induced the phosphorylation of RSK2 at both Ser227 and Thr577 (Fig. 2A). Next, we assessed the activation of Atm in RSK2 WT and KO MEFs treated with AD. We observed phosphorylation of Atm at Ser1981 in RSK2 WT MEFs starting at 2 hr after AD treatment, indicating the presence of DNA DSBs. However, the phosphorylation of Atm at Ser1981 was reduced in RSK2-deficient MEFs compared to the WT cells (Fig. 2A).

p53 is one of the most important effector molecules downstream of Atm in the DNA damage response. p53 phosphorylation induced by DNA damage either stabilizes p53 or enhances its transactivation ability. We therefore investigated the phosphorylation of p53 in both RSK2 WT and KO MEFs. Western blot analysis revealed that p53 was phosphorylated at Ser18 (human Ser15) in WT cells. RSK2 deficiency led to drastically reduced phosphorylation at Ser18, as well as a reduction in p53 up-regulation in response to AD (Fig. 2A). p53 is a master gene that regulates cell cycle progression, apoptosis, and DNA repair in cells with DNA damage. Decreased phosphorylation of p53 at Ser18 indicates that downstream target genes that control these cellular events might be affected. Indeed, we found that while AD treatment of WT cells leads to an induction of p21, a potent cyclin-dependent kinase inhibitor (CDKI) that functions as a regulator of cell cycle progression at the G1 phase [22], this effect was abolished in RSK2 KO cells (Fig. 2A). We further observed that RSK2 deficiency had no effect on the expression levels of Bax, a molecule that is involved in p53-mediated apoptosis [23], compared to WT cells, suggesting that RSK2 plays a critical role in DNA damage-induced p53 activation and cell cycle progression. To further confirm the effects of the RSK2-mediated DNA damage response, we overexpressed RSK2 in RSK2-deficient MEFs and observed that the disrupted phosphorylation of Atm at Ser1981 and p53 at Ser18 in these cells was rescued by RSK2 overexpression (Fig. 2B).

To further confirm this finding, we analyzed CLS fibroblasts obtained from patients with RSK2 mutations. Genotoxic stress-induced RSK2 phosphorylation at Ser227 and Thr577 was observed in healthy human fibroblasts (Fig. 2C). Notably, however, we observed a decreased phosphorylation of Atm at Ser1981 in CLS fibroblasts (Fig. 2C). Furthermore, p53 phosphorylation at Ser15 was abolished in the RSK2-deficient CLS cells, as well as downstream p21 signaling. However, Bax activity was not affected. Taken together, our findings from the RSK2 KO mouse cell line and the RSK2-deficient human cell line indicate that RSK2 activates the Atm/p53-dependent DNA damage pathway.

### Genotoxic stress promotes RSK2 interaction with Atm

**Figure 3:**
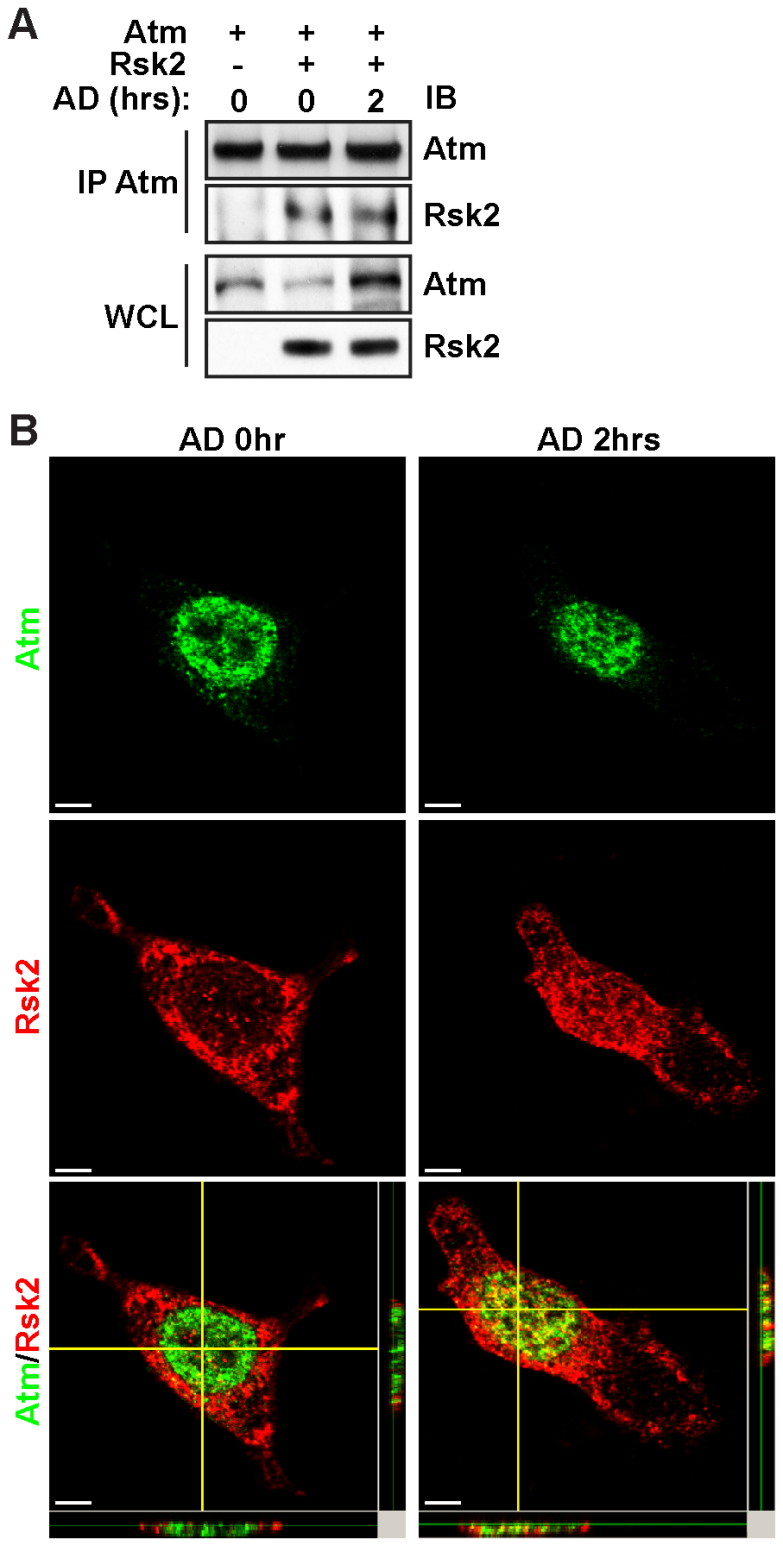
Genotoxic stress promotes the interaction of RSK2 with Atm. A. Ectopically expressed RSK2 interact with Atm, and the formation of this complex is enhanced under conditions of genotoxic stress. NIH cells were transfected with the indicated expression constructs and treated with 0.5 µM AD 2 hr or left untreated. Atm was then immunoprecipitated from the cell lysates. The immunoprecipitated proteins and their associated proteins were detected by western blot analysis using anti-Atm, -RSK2 antibodies, respectively. B. Under conditions of genotoxic stress, activated RSK2 translocated to the nucleus and co-localized with Atm. MEF cells were co-transfected with Atm and RSK2 and then treated with 0.5 µM AD for 2 hr. The cells were fixed, permeabilized, and blocked, and immunocytochemistry was performed using primary antibodies against RSK2 and Atm and FITC-conjugated or Alexa Fluor 555 Donkey anti-mouse IgG (H+L) secondary antibodies. Scale bars: 5 µm. A representative Z-stack image shows RSK2 and Atm double-positive cells.

To further define the mechanism of how RSK2 activates the Atm-dependent DNA damage pathway, we next sought to determine whether RSK2 and Atm interact with each other under conditions of genotoxic stress. RSK2 shuttles between the cytoplasm and the nucleus upon activation [24]. Co-immunoprecipitation assays showed that upon overexpression of RSK2 and Atm in NIH cells, Atm associates with RSK2. Importantly, this interaction was further enhanced by genotoxic stress induced by AD treatment for 2 hr (Fig. 3A). To confirm this result, we co-transfected RSK2 and Atm into MEFs, which were then immunostained for both proteins. Under normal conditions, RSK2 is predominantly in the cytoplasmic compartment, whereas Atm is mainly nuclear. Thus, there was little co-localization between these two proteins (Fig. 3B). However, after AD administration for 2 hr, enhanced nuclear co-localization of RSK2 and Atm was observed (Fig. 3B). These results suggest that Atm interacts with RSK2 under conditions of genotoxic stress.

### RSK2 deficiency relieves genotoxic stress-induced G2/M cell cycle arrest

**Figure 4:**
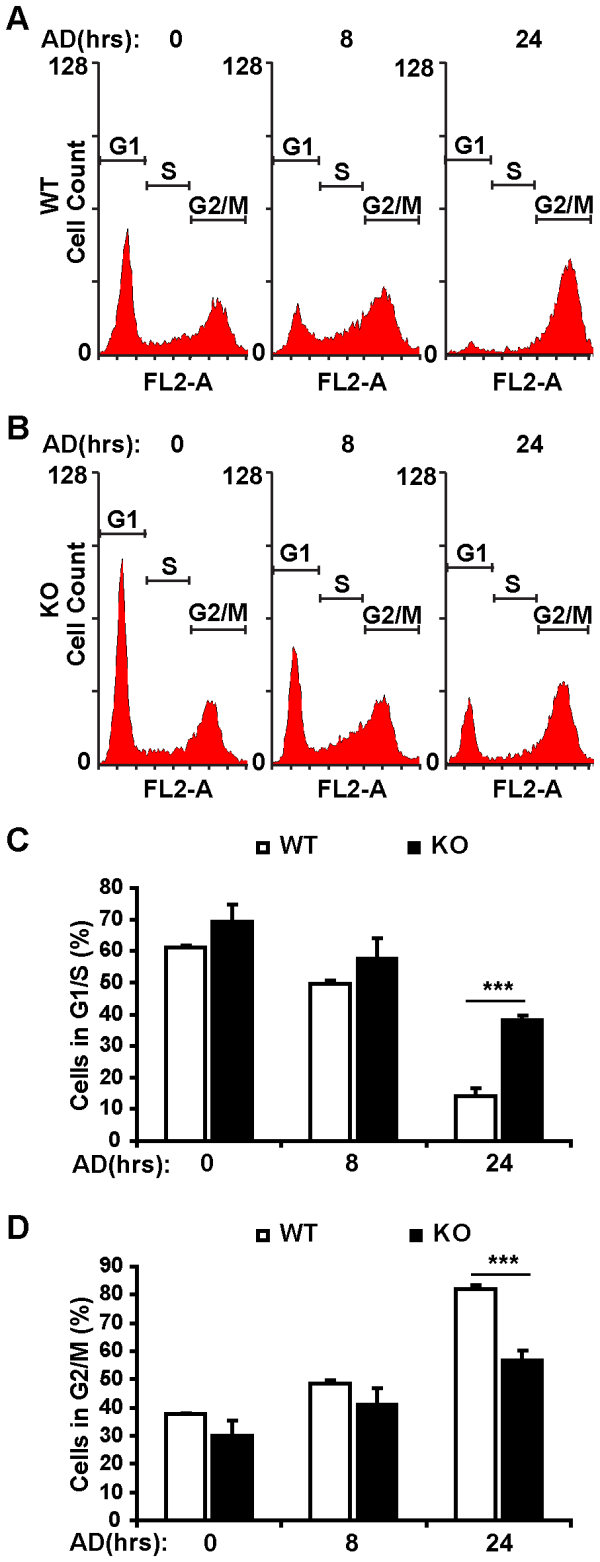
RSK2 regulates cell cycle progression in response to genotoxic stress. A and B. Cell cycle profile analysis using FACs. RSK2 WT (A) and KO (B) MEFs were treated with 0.5 µM AD at various time points, and the cells were fixed and stained with propidium iodide, followed by flow cytometry analysis. C. At 24 hr, an increased percentage of RSK2 KO MEFs had entered the G1/S phase compared to WT MEFs. D. At 24 hr, a decreased number of RSK2 KO MEFs had entered the G2/M phase compared to WT MEFs. For each experiment, at least three independent replicates were performed, and similar results were obtained. Representative results from one experiment are shown. All data were shown as the mean plus or minus the standard deviation of the mean (mean ± SD). A significant difference was defined as ***P<0.001 compared to WT cells.

DNA damage typically results in three cellular responses: cell cycle arrest, increased DNA repair, and apoptosis [25]. Previous studies have shown that RSK2 is mainly involved in proliferation and survival upon exposure to UVB or an AD analogue [17], [26]. In addition, our results also suggested that in response to DNA damage, RSK2 primarily affects p21, which is involved in cell cycle regulation rather than apoptosis (Fig. 2). Hence, we examined the impact of RSK2 deficiency on cell cycle arrest under genotoxic stress conditions for 8 hr and 24 hr. We performed flow cytometry analysis of AD-treated RSK2 KO and WT MEFs. Propidium Iodide (PI) staining showed approximately 40% of control (untreated) cells were in G2/M phase (Fig. 4A, 4B, 4D). Treatment of cells with AD increased this number to 80%, indicating AD-induced G2/M arrest (Fig. 4D). Interestingly, reduced percentage of cells in the G2/M phase (58%) was observed in the RSK2 KO cells (Fig. 4D). Moreover, recovered number of cells in the G1/S phase was detected in the RSK2 KO cells (Fig. 4C). These data indicate that RSK2 deficiency relieves AD-induced G2/M arrest, suggesting that RSK2 plays a critical role in genotoxic stress-induced cell cycle progression.

### RSK2 deficiency increases vulnerability of the cells to genotoxic stress

**Figure 5:**
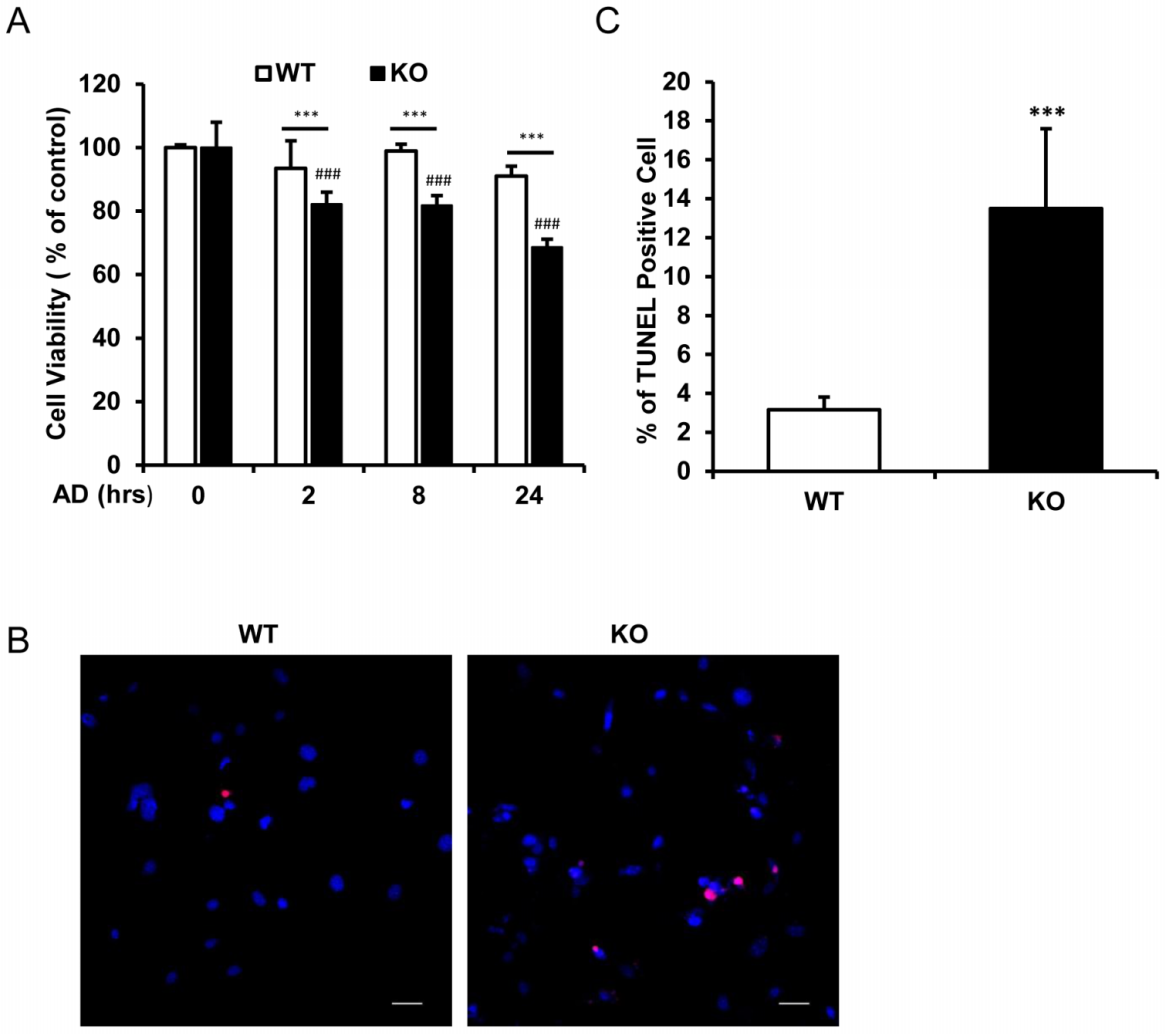
RSK2 deficiency increases cell vulnerability to genotoxic stress. A. WST1 assay indicated the cell viability decreases in RSK2 WT and KO MEFs treated with AD (0.5 µM) at indicated time scale (0–24 h). n≥8. ***P<0.001 compared to WT cells at same time point. ^###^P<0.001 compared to 0 h control. B. TUNEL assay indicated the percentage of dead cells after 24 h AD (0.5 µM) treatment. Representative image of TUNEL assay for cell death in RSK2 WT and KO MEFs treated with AD. Scale bar indicated 50 µm. C. Grouped result showing the statistics of TUNEL assay in RSK2 WT and KO MEFs n≥5. ***P<0.001 compared to WT cells.

As impaired DNA repair would lead to cell apoptosis, we also test the cell viability in RSK2 WT and KO cells treated with Adriamycin (0.5 µM). As shown in Fig. 5A, Adriamycin treatment significantly decreased cell viability in RSK2 KO cells but not in RSK2 WT cells. Compared with RSK2 WT cells, RSK2 KO cells displayed lower cell viability at each time point after treatment. In addition to that, we also performed TUNEL assay to detect the cell death effect under the genotoxic stress. RSK2 KO cells exhibited more cell death than RSK2 WT cells after 24 h Adriamycin treatment (Fig. 5B, C). Our results suggested that RSK2 deficiency increased vulnerability of cells to genotoxic stress.

### RSK2 deficiency results in an increased number of γH2AX foci in response to genotoxic stress

**Figure 6:**
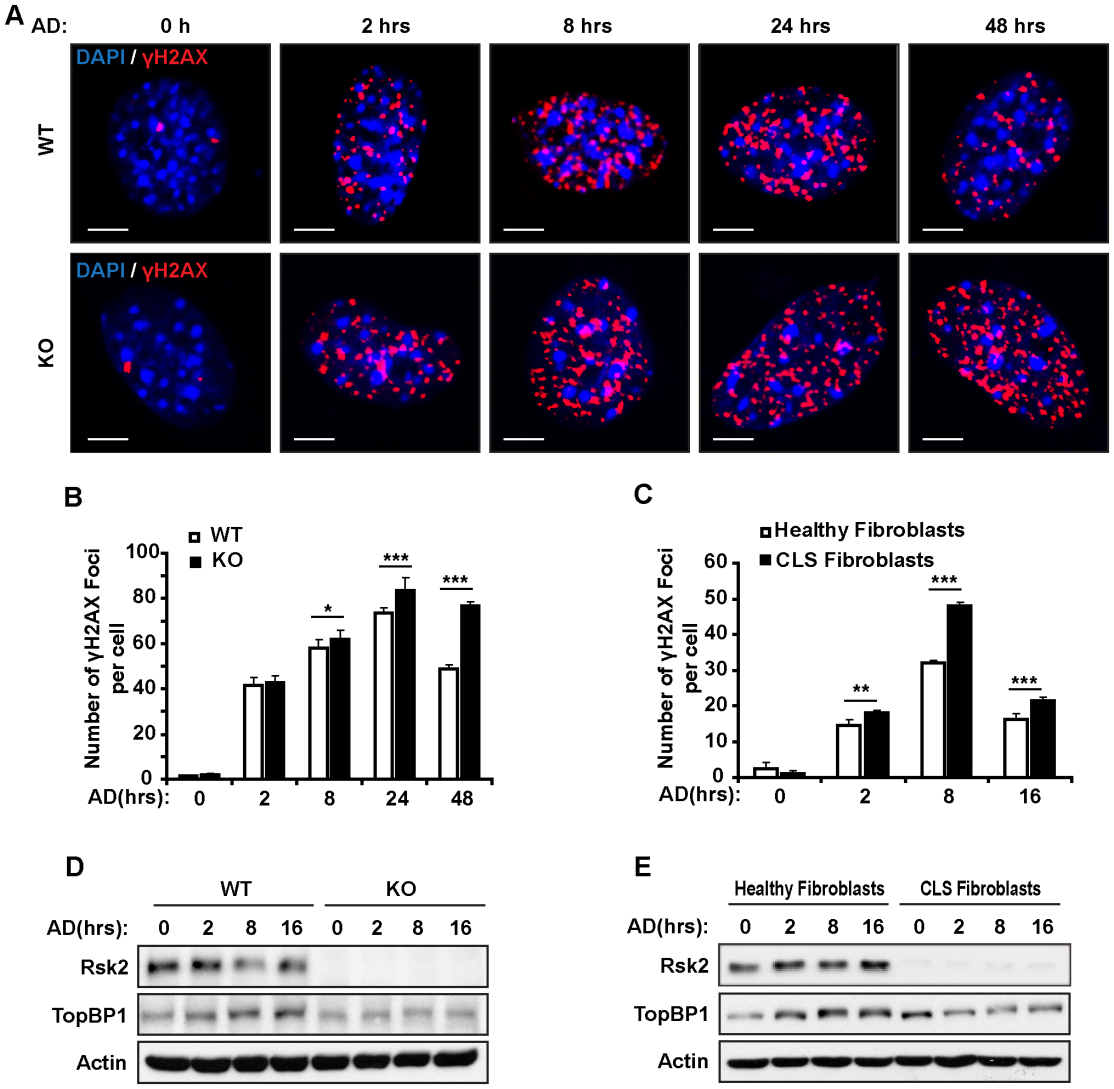
RSK2 regulates DNA repair efficiency under conditions of genotoxic stress. A. Increased formation of AD-induced γH2AX foci in RSK2 KO MEFs. RSK2 KO and WT MEFs were exposed to 0.5 µM AD for different periods of time from 2 hr to 48 hr, and then immunostained for endogenous γH2AX. Scale bars: 10 µm. B. Quantitation of the data from image (A) showing the number of γH2AX foci per cell in RSK2 WT and KO MEFs. RSK2 KO cells showed a significant increase in the number of γH2AX foci at 8 hr, 24 hr, and 48 hr, compared to WT cells. C. Similar to RSK2 KO cells, RSK2-deficient fibroblasts from CLS patients displayed an increased number of γH2AX foci compared to fibroblasts from healthy controls at 2 hr, 8 hr, and 16 hr. D. RSK2 KO MEFs show decreased expression levels of TopBP1, compared to WT MEFs. RSK2 WT and KO MEFs were treated with 0.5 µM AD treatment for 2 hr, 8 hr, and 16 hr, follow by western blot analysis with an antibody against TopBP1. The expression level of TopBP1 in RSK2 WT MEFs increased in response to 0.5 µM AD treatment, but this response was impaired in RSK2 KO MEFs. E. Similarly, TopBP1 was down-regulated in response to AD in the CLS patient fibroblast cells compared to cells healthy control. For each experiment, at least three independent replicates were performed, and similar results were obtained. Representative results from one experiment are shown. All data are shown as the mean plus or minus the standard deviation of the mean (mean ± SD). A significant difference was defined as *P<0.05, **P<0.01, and ***P<0.001 compared to WT cells.

When the cell cycle is arrested, specific DNA repair mechanisms are activated depending on the types of DNA lesions and the cell cycle phase [27]. We next examined the potential role of RSK2 in DNA repair under genotoxic stress conditions. Proteins that are responsible for the DNA damage response and DNA repair are rapidly recruited to the sites of breaks, leading to the formation of nuclear foci. These foci disappear upon completion of DNA damage repair [28]. We therefore investigated whether the AD-induced formation of γH2AX foci is affected by RSK2. γH2AX is phosphorylated at Ser139 by various kinases and forms foci at the sites of DSBs [29]–[31]. Due to this localization pattern, γH2AX foci are commonly used to quantify the severity of DSBs and the efficiency of DNA repair, with a high number of foci indicating a deficiency in DNA repair. We treated RSK2 WT and KO MEFs with AD for different periods of time (from 2 hr to 48 hr) and quantified the average number of γH2AX foci per cell by immunostaining the cells for endogenous γH2AX (Fig. 6A). Untreated WT and KO MEFs formed very few foci (∼1–2 foci/cell). After 2 hr of treatment with 0.5 µM AD, RSK2 WT and KO MEFs started to form foci, but no difference was detected between the two groups (Fig. 6A and 6B). We observed an increase in the number of foci per cell in both RSK2 WT and KO MEFs over time, which reached a peak at 24 hr (Fig. 6B: WT-75 foci/cell, and KO-84 foci/cell). Interestingly, we observed an increase in the number of foci per cell at 8 hr (61 foci/cell), 24 hr (84 foci/cell), and 48 hr (75 foci/cell) in the RSK2 KO group compared to the WT cells (which showed 55 foci/cell, 75 foci/cell, and 48 foci/cell at these time points, respectively) (Fig. 6A and B). The significant difference in the number of γH2AX foci observed over time (from 8 to 48 hr) suggests that RSK2 deficiency might cause a delay in DNA repair, resulting in the persistently high number of γH2AX foci in RSK2-deficient MEFs.

To determine whether DNA repair efficiency is impaired in CLS, we treated healthy human fibroblasts and CLS patient fibroblasts with AD and measured the number of γH2AX foci per cell. We observed an increase in γH2AX foci formation for both groups beginning at 2 hr of AD treatment, which reached a peak at 8 hr, followed by a reduction in the number of foci number by 16 hr. However, CLS fibroblasts displayed a significantly higher number of foci per cell at 2 hr (18 foci/cell), 8 hr (48 foci/cell), and 16 hr (21 foci/cell) of AD treatment, compared to healthy fibroblasts (which showed 13 foci/cell, 32 foci/cell, and 16 foci/cell at these time points, respectively) (Fig. 6C). Hence, the results from human RSK2 mutant cell lines were consistent with our findings in RSK2-deficient mouse cells (Fig. 6A and 6B), indicating that the lack of functional RSK2 leads to deficiency in DNA repair.

We next analyzed TopBP1, a nuclear protein that localizes at the sites of DNA DSBs and affects DNA repair [32]–[33]. Notably, western blot analysis demonstrated that the levels of TopBP1 decreased in RSK2 KO MEFs in response to AD treatment, compared to WT cells (Fig. 6D). TopBP1 was also down-regulated in the CLS patient fibroblast cells (Fig. 6 E). Thus, the decreased level of TopBP1 in RSK2-deficient cells indicates that RSK2 is important in maintaining DNA repair efficiency.

## Discussion

**Figure 7:**
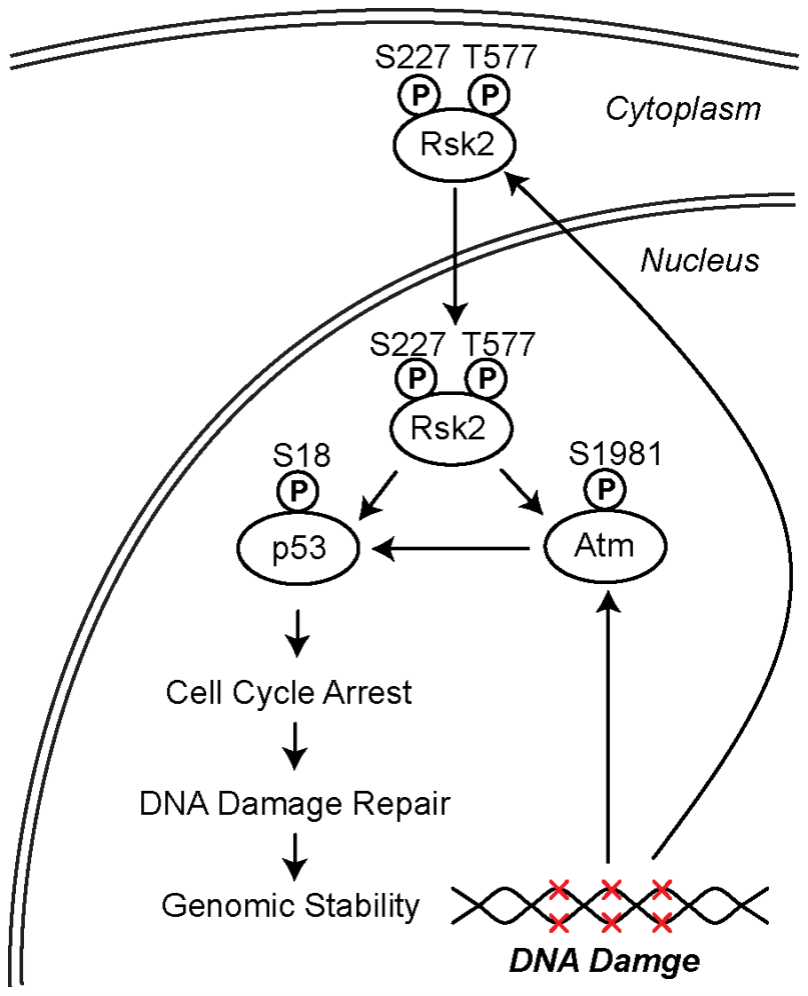
RSK2 maintains genome stability in response to genotoxic stress. RSK2 is phosphorylated at both Ser227 and Thr577 in response to DNA damage. Activated RSK2 translocates to the nucleus and interacts with Atm, thus activating the Atm/p53-dependent DNA damage pathway, as well as p53-controlled cellular events. RSK2 KO relieves AD-induced cell cycle arrest at the G2/M check point and subsequently inhibited the DNA repair process, as indicated by the increased formation of γH2AX foci.

Increasing evidence suggests that RSK2 is involved in DNA damage-induced signaling pathways. For instance, RSK2 is activated by UV irradiation, which causes DNA strand breaks, and this activation transduces signals to specific downstream effectors, such as RNA-activated Protein Kinase (PKR) and Bcl-2-Associated Death promoter (BAD) [34]–[35]. However, the physiological function of RSK2 in the DNA damage response and DNA repair has not been well elucidated. Here, we show that RSK2 is phosphorylated at both Ser227 and Thr577 in response to genotoxic stress. We also show that RSK2 activates the Atm/p53-dependent DNA damage pathway by interacting with Atm, which acts as a sensor for DNA damage. We further showed that RSK2 KO relieves AD-induced cell cycle arrest at the G2/M check point and subsequently inhibits the DNA repair process by reducing the formation of γH2AX foci (Fig. 7).

Our results suggested that RSK2 was activated under genotoxic stress. As shown in Fig. 1, in MEF cells treated with adriamycin, the phosphorylation of RSK2 was elevated and accumulated in nuclear. It has been reported that RSKs were activated via ERK and JNK pathway under UV-irritation [26], [36], which suggested a possible signaling mechanism for RSK2 activation in cells with genotoxic stress. Further investigation will be carried out to elucidate the underlying mechanisms.

RSK2 does not contain a classic nuclear localization signal (NLS), and the mechanism by which it translocates to the nucleus has not been investigated previously. In response to mitogen treatment, RSK2 is slowly released from stress granules and shuttles rapidly in and out of the nucleus [37]. Several studies have suggested that the phosphorylation status of RSK is important for its nuclear translocation [38]. Other studies have also indicated that various binding partners regulate the subcellular distribution of RSK [39]–[40]. ATM is the key player in DNA damage response, which is predominantly present within the nucleus of cultured human cells, with a small fraction present in the cytoplasm [41]. Our results demonstrated that RSK2 deficiency impaired ATM function under genotoxic stress. As suggested in Fig. 2, RSK2 KO cells and CLS Fibroblast with RSK2 mutants displayed a dysfunction of ATM activation. Overexpression of RSK2 was able to rescue the ATM phosphorylation levels but not ATM protein levels (Fig. 2B), indicating that RSK2 might be essential for ATM activation. Moreover, we found that cytosolic RSK2 translocates to the nucleus when DNA damage occurs. As Atm is predominantly expressed in the nucleus, transport of RSK2 to the nucleus enables the formation of a RSK2-Atm complex, promotes the activation of ATM and facilitates the Atm/p53-dependent DNA damage pathway in response to DNA damage. Atm, Atr, and DNA-PKs are DNA damage-sensing protein kinases that signal the presence of DNA lesions, initiate cell cycle arrest, and DNA repair or apoptosis through a series of phosphorylation events [27].

We postulate that the phosphorylation of RSK2 is required to fully activate Atm at Ser1981 and p53 at Ser18 (mouse) or Ser15 (human) in response to genotoxic stress. Atm has long been known to phosphorylate p53 at Ser15, promoting p53 accumulation and activation in response to DNA damage [42]. Previous studies have indicated that p53 is another important substrate of RSK2 for chromatin remodeling and regulation of gene expression. RSK2 activates and phosphorylates p53 (Ser15) *in vitro* and *in vivo* and co-localizes with p53 in the nucleus [17]. Upon UVB stimulation, phosphorylation of p53 at Ser15 in cells from CLS patients lacking RSK2 was noticeably reduced compared to p53 phosphorylation in healthy cells, showing a crucial role for RSK2 in p53 activation in response to DNA damage. As p53 is a common target for both Atm and RSK2 in the presence of DNA lesions, this suggests the existence of a DNA damage pathway that involves both RSK2 and Atm. In addition, our analysis also showed that RSK2 interacts with Atm shortly after the induction of DNA damage, while Atm activation is greatly diminished when RSK2 is absent or mutated (Fig. 2). Quantification of γH2AX foci revealed that DNA repair ability is reduced in Atm-defective human fibroblasts treated with Neocarzinostatin, a radiomimetic reagent that induces DSBs [43]. This observation correlates well with our observation that RSK2 KO MEFs and CLS fibroblasts exhibit a significantly higher number of γH2AX foci per cell with longer periods of AD treatment, indicating deficiencies in DNA repair in RSK2-deficient cells. These findings demonstrate that both RSK2- and Atm-deficient cells display similar functional defects, presumably meditated by their common downstream target p53. Thus our study suggests that RSK2 plays an important role in the Atm/p53-dependent DNA damage pathway and DNA repair.

RSK2 does not contain a classic nuclear localization signal (NLS), and the mechanism by which it translocates to the nucleus has not been investigated previously. In response to mitogen treatment, RSK2 is slowly released from stress granules and shuttles rapidly in and out of the nucleus [37]. Atm is predominantly present within the nucleus of cultured human cells, with a small fraction present in the cytoplasm [41]. Several studies have suggested that the phosphorylation status of RSK is important for its nuclear translocation [38]. Other studies have also indicated that various binding partners regulate the subcellular distribution of RSK [39]–[40]. Here, we found that cytosolic RSK2 translocates to the nucleus when DNA damage occurs. As Atm is predominantly expressed in the nucleus, transport of RSK2 to the nucleus enables the formation of a RSK2-Atm complex and activates the Atm/p53-dependent DNA damage pathway in response to DNA damage. Atm, Atr, and DNA-PKs are DNA damage-sensing protein kinases that signal the presence of DNA lesions, initiate cell cycle arrest, and DNA repair or apoptosis through a series of phosphorylation events [27].

Impaired DNA damage response would promote apoptosis [44]. We also observed that RSK2 KO cells exhibited decreased cell viability and elevated cell death under genotoxic stress compared to RSK2 WT cells (Fig. 5 A and B). However, we did not observe alternations of Bax expression in both RSK2 WT and KO cells. The expression of Bax is regulated by p53 and will be elevated under apoptotic signals [45]. We postulated that in our experiments, the Adriamycin treatment (0.5 µM) in immortalized MEF cells was able to induce p53 signaling to cell cycle arrest but not severe enough to induce massive apoptosis. Therefore we found that p21 but not Bax was up-regulated during genotoxic stress.

In summary, our study has uncovered a novel role for RSK2 in a DNA damage and repair pathway that maintains genomic stability by regulating cell cycle progression and DNA repair. We believe that the elucidation of this regulatory network has greatly enhanced our understanding of the underlying causes of cognitive dysfunction observed in CLS. In addition, our results confirm the link between CLS, a neurological disorder caused by mutated RSK2, and defects in the DNA damage response.

## Materials and Methods

### Animals

All C57BL/6 mice were maintained in accordance with the institutional guidelines, and all protocols were approved through the Institutional Animal Care and Use Committee (IACUC) of the National Neuroscience Institute, Tan Tock Seng Hospital. The mice were maintained in a pathogen-free facility and exposed to a 12 h light/dark cycle with food and water.

### Cell culture and transfection

Low passage (p3–p5) mouse embryonic fibroblasts (MEFs) were prepared as previously described [46]. MEFs were cultured in DMEM supplemented with 10% FCS and penicillin/streptomycin in an atmosphere of 5% CO_2_ at 37°C. Human CLS (GM09621) and healthy fibroblast cells (GM03321) were purchased from Coriell Cell Repositories. The RSK2 knockout MEFs were provided by Dr. R. Strachan, Duke University [47]. The RSK2 DNA constructs (imaGenes, GmbH) and Atm DNA constructs (a kind gift from Dr. XY. Wang, the National University of Singapore) were transfected into NIH cells and MEFs using lipofectamine (Invitrogen) according to manufacturer's protocol.

### Genotoxic stress and antibodies

DNA damage was generated by inducing genotoxic stress with 0.5 µM Adriamycin (doxorubicin) (CalBiochem, USA). Antibodies against RSK2 (#SC-9986), Ser227 RSK2 (#SC12445), Thr577 RSK2 (#SC-16407), and actin (#SC-69879) were purchased from Santa Cruz; antibodies against p53 (#2524) and Ser15 p53 (#9284) were purchased from Cell Signaling; antibodies against Bax (#554104), p21 (#556430), and TopBP1 (#611875) were purchased from BD Biosciences; the antibody against Atm (GTX70103) was purchased from GeneTex; and the antibody against Ser1981 Atm (200-301-400) was purchased from Rockland Immunochemicals.

### Chromatin Fractionation

MEF cells were treated with Adriamycin (AD) and harvested at specific time points. Chromatin fractionation was performed as described previously [21]. In brief, cells were incubated with Buffer A containing 10 mM HEPES, 10 mM KCl, 1.5 mM MgCl_2_, 0.34 M Sucrose, 10% Glycerol, 1 mM DTT, and a mixture of protease and phosphatase inhibitors for 5 min and then lysed. Triton X-100 was added to a final concentration of 0.1%. Lysed cells were incubated on ice for 4 min and then centrifuged at 1300 rcf for 4 min at 4°C. The supernatant (S1) and pellet (P1) fractions were collected. The S1 fraction was further centrifuged at 1600 rcf for 15 min at 4°C, and the supernatant (S2) was collected. The P1 fraction was washed once with Buffer A and lysed for 30 min on ice with Buffer B, which contained 3 mM EDTA, 0.2 mM EGTA, and a mixture of protease and phosphatase inhibitors. The lysed P1 fraction was centrifuged at 1700 rcf for 4 min at 4°C. The resulting pellet (P3) was washed once with Buffer B and re-suspended in Laemmli buffer. The S2 (cytosolic proteins) and P3 (chromatin associated proteins) fractions were then analyzed by SDS-PAGE and western blot.

### Immunoprecipitation and western blot

After drug treatment, cells were washed with PBS and lysed in RIPA buffer containing phosphatase and protease inhibitors. The protein concentration was determined by Bio-Rad assay. Equal amounts of protein (15 µg) were separated by electrophoresis on SDS–PAGE gels and then transferred onto nitrocellulose membranes (Millipore), which were probed with primary and secondary antibodies, and visualized using an ECL kit (GE Healthcare). For immunoprecipitation, antibodies were added to the cell lysates and incubated at 4°C overnight, followed by incubation with Protein A plus G beads at 4°C for 4 hr. The immunoprecipitated proteins were released from the beads by boiling in 2× sample buffer for 5 min and subsequently analyzed by western blot.

### FACs analysis

After drug treatment, cells were trypsinized with 0.05% trypsin and 0.5 mM EDTA (Gibco BRL) at 37°C for 2 min and collected by centrifugation at 1000 rpm for 5 min. For cellular DNA content analyses, cells were then washed once in PBS and subsequently fixed in 70% ethanol at −20°C overnight. The ethanol solution was then removed, and the cells were washed once with PBS containing 5 mM EDTA. The cells were then re-suspended in hypotonic Propidium Iodide (PI) buffer for 3 hr in the dark at room temperature as described previously [48]. Counts of PI-stained cells were immediately acquired using an LSRII flow cytometer (Becton and Dickinson Biosciences) and analyzed using WinMDI Version 2.9 software (The Scripps Research Institute, La Jolla, CA, USA).

### WST-1 assay

WST-1 assay kit (Roche, Switzerland) was used to test cell viability. Both the RSK2 WT and KO MEFs were seeded equally into 96-well plate. The cells were treated with AD (0.5 µM) for time periods as indicated (2–24 h), followed by incubation with WST-1 (1∶10) for additional 2 h. The absorbance at a wavelength of 450 nm was measured by the microplate reader (Tecan, Austria).

### TUNEL assay

The *in situ* cell death detection kit (Roche, Switzerland) was used for detecting cell injury after AD treatment. Both the RSK2 WT and KO MEFs were seeded on cover slips. After treatment of Adriamycin for 24 h, the cells were fixed in 4% paraformaldehyde for 1 h at room temperature. Following fixation, the cells were rinsed with PBS and permeabilized with 0.1% Triton X-100 in 0.1% sodium citrate on ice for 2 min. Then the cells were incubated at 37°C with TUNEL reaction mixture for 1 h. The TUNEL positive cells were visualized on a confocal microscope (Olympus, FV-10).

### Immunocytochemistry

After drug treatment, cells were fixed, permeabilized with 0.1% Triton X-100, and blocked with 2% BSA. Primary antibodies against γH2AX (A300-081A, Bethyl Lab), Atm (SC-7230, Santa Cruz), and RSK2 (SC-9986, Santa Cruz) were added, followed by incubation with Alexa Fluor 555 Goat Anti-Rabbit IgG (H+L) (Invitrogen, A21428), Alexa Fluor 488 Goat Anti-Rabbit IgG (H+L) (Invitrogen, A11043), and Alexa Fluor 555 Donkey Anti-Mouse IgG (H+L) (Invitrogen, A31570) secondary antibody. The slices were mounted with mounting medium containing DAPI and visualized on a confocal microscope (Olympus, FV-10).

### Image acquisition, western blot quantification, and statistical analysis

For each experiment, at least 3 independent replicates were performed, and similar results were obtained. Representative results from one experiment are shown. For quantification, all western blots were scanned with a Molecular Dynamics scanning densitometer. Statistical analysis was performed using ANOVA and Student's *t* test. All data are shown as the mean plus or minus the standard deviation of the mean (mean ± SD). A significant difference was defined as *P<0.05, **P<0.01, and ***P<0.001 compared to the control.
